# Inhibition of astrocyte metabolism is not the primary mechanism for anaesthetic hypnosis

**DOI:** 10.1186/s40064-016-2734-z

**Published:** 2016-07-11

**Authors:** Logan J. Voss, Martyn G. Harvey, James W. Sleigh

**Affiliations:** Anaesthesia Department, Waikato District Health Board, Pembroke St, Hamilton, 3240 New Zealand; Emergency Department, Waikato District Health Board, Hamilton, 3240 New Zealand; University of Auckland Waikato Clinical School, Hamilton, 3240 New Zealand

**Keywords:** Anaesthesia, Hypnosis, Astrocyte, Glia, Cortical slice

## Abstract

Astrocytes have been promoted as a possible mechanistic target for anaesthetic hypnosis. The aim of this study was to explore this using the neocortical brain slice preparation. The methods were in two parts. Firstly, multiple general anaesthetic compounds demonstrating varying in vivo hypnotic potency were analysed for their effect on “zero-magnesium” seizure-like event (SLE) activity in mouse neocortical slices. Subsequently, the effect of astrocyte metabolic inhibition was investigated in neocortical slices, and compared with that of the anaesthetic drugs. The rationale was that, if suppression of astrocytes was both necessary and sufficient to cause hypnosis in vivo, then inhibition of astrocytic metabolism in slices should mimic the anaesthetic effect. In vivo anaesthetic potency correlated strongly with the magnitude of reduction in SLE frequency in neocortical slices (R^2^ 37.7 %, p = 0.002). Conversely, SLE frequency and length were significantly enhanced during exposure to both fluoroacetate (23 and 20 % increase, respectively, p < 0.01) and aminoadipate (12 and 38 % increase, respectively, p < 0.01 and p < 0.05). The capacity of an anaesthetic agent to reduce SLE frequency in the neocortical slice is a good indicator of its in vivo hypnotic potency. The results do not support the hypothesis that astrocytic metabolic inhibition is a mechanism of anaesthetic hypnosis.

## Background

 Understanding how anaesthetics cause loss of consciousness (hypnosis) is of critical importance to both clinical anesthesiologists and neuroscientists—for both safer clinical application and understanding the biological basis of consciousness. Direct effects on neuronal targets are assumed to be involved, however it is interesting to note that glia have also been shown to be important targets for anaesthetic drugs (Jevtovic-Todorovic et al. 2013; Rath et al. 2008; Schummers et al. 2008; Thrane et al. 2012). In particular, Thrane et al. (2012) have recently shown that a chemically diverse group of anaesthetics inhibit astrocytic calcium signalling—the basis of astrocytic glutamatergic regulation of neuronal synaptic activity (Parpura and Haydon 2000). The implication is that the hypnotic effect of anaesthetics may be due to direct inhibitory effects on non-neuronal astrocytic networks.

In vivo models are not ideally suited for disentangling anaesthetic hypnotic mechanisms because of the challenge of isolating the effects of interest in a controlled fashion. In vitro models on the other hand offer the advantage that experimental conditions can be manipulated and controlled at will. The isolated brain slice preparation is a case in point and has been used extensively to investigate mechanisms of anaesthetic effect (Antkowiak and Heck 1997; Becker et al. 2012; Ries and Puil 1999; Ying et al. 2006). In particular, a range of chemically distinct anaesthetics have been shown to have repeatable and robust effects on neocortical field potential dynamics (Voss et al. 2012, 2013). In this study we have taken advantage of these effects to investigate whether a disruption to astrocytic networks can explain the functional anaesthetic end-point of hypnosis.

The study was carried out in two parts. Firstly, we utilised a range of chemically related ketamine-ester analogues shown to have widely varying in vivo hypnotic potencies—and identified in cortical slices the field potential correlates of the in vivo hypnotic effect. We found that the in vivo hypnotic potency of the ketamine-esters correlated with their ability to reduce the frequency of zero-magnesium seizure-like events (SLEs) in the slice. Propofol and etomidate—drugs which are thought to act on a different family of receptors to ketamine—were also shown to have dose-dependent inhibitory effects on SLE frequency, in dose ranges consistent with their relative clinical potencies for inducing hypnosis. This is in agreement with previous studies (Voss et al. 2012) and indicates that a reduction in SLE frequency is the pathognomonic signature of anaesthetic hypnotic action in cortical brain slices. With this as a basis, we hypothesised in the second part of this paper that if astrocyte inhibition is sufficient to cause hypnosis during anaesthesia, then inhibition of astrocyte function in the cortical slice model should result in a decrease in SLE frequency.

## Results and discussion

### Part 1 results: correlating in vivo hypnotic potency with cortical slice electrophysiology

**Fig. 1:**
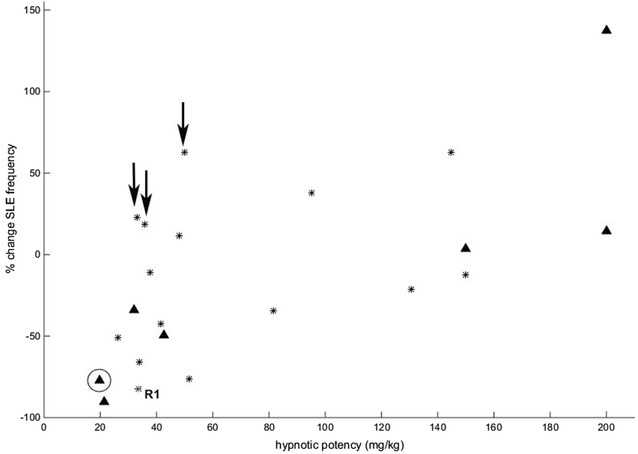
Scatterplot of the change in seizure-like event (SLE) frequency against in vivo hypnotic potency (mg/kg) for ketamine and each of the 21 ketamine analogues. *Triangles* are the non-ester analogues (including ketamine—*circled*); *stars* are the ester analogues. The three agents identified by *arrows* that were moderately potent hypnotics but did not induce a reduction in SLE frequency were either seizureogenic or had very rapid offset (<100 s)

**Fig. 2:**
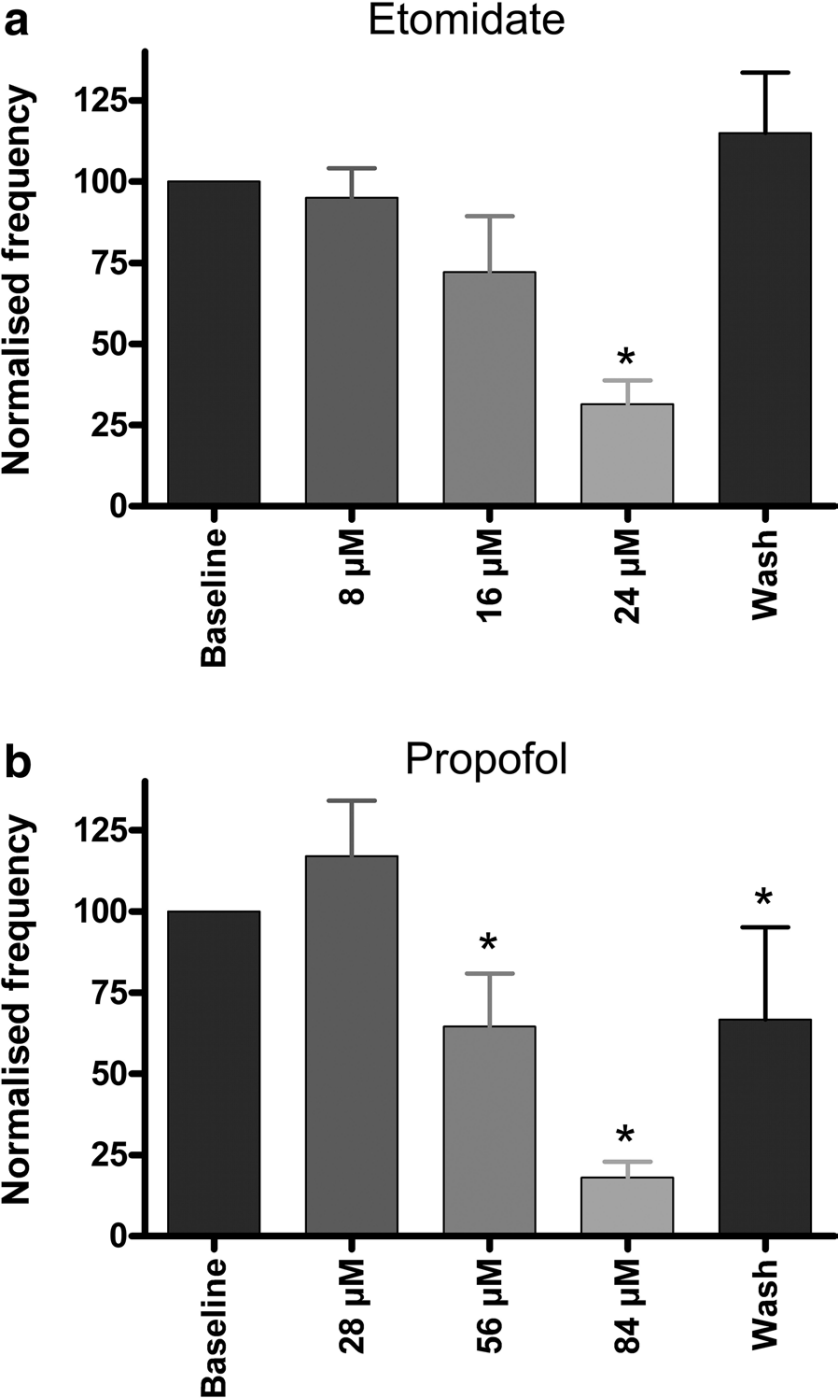
*Graphs* showing the effect of sequentially higher doses of **a** etomidate (n = 18) and **b** propofol (n = 28) on the frequency of seizure-like event frequency. Each dose was perfused for 30 min or until SLE frequency reduced by at least 50 % compared to the baseline value. The data are normalised to the baseline frequency and are expressed as mean + SEM. *p < 0.001, compared to baseline, Friedman Test with Dunn’s Multiple Comparisons

A large range of hypnotic potencies were represented in the suite of ketamine-ester analogues, matched by equally variable slice SLE responses. Figure 1 plots the change in SLE frequency against in vivo hypnotic potency and shows a strong positive correlation (R^2^ 37.7 %, p = 0.002)—indicating that the more potent hypnotics induced larger reductions in event frequency. The correlation was strongly driven by the non-ester analogues (R^2^ = 73 %, p = 0.01, n = 7), confirming that the relationship was based largely on drug pharmacodynamics, not the kinetics of ester break-down. Neither change in SLE length, nor amplitude correlated significantly with hypnotic potency (R^2^ 5.7 and 0.3 %, respectively). The dose-dependent effect of propofol and etomidate on SLE frequency (Fig. 2) further indicates that the ability of an agent to reduce SLE frequency in the cortical slice is a good indicator of its hypnotic capacity in vivo. This is consistent with previous investigations showing that clinically used anaesthetics have in common the capacity to strongly reduce SLE frequency in cortical slices (Voss and Sleigh 2010; Voss et al. 2012). We reasoned that if inhibitory effects on astrocytes underpinned the ability of anaesthetics to induce hypnosis, then a measurable reduction in SLE frequency should be evident following blockade of astrocytic metabolism in the cortical slice.

### Part 2 results: effect of astrocyte inhibition on cortical slice SLE activity

**Fig. 3:**
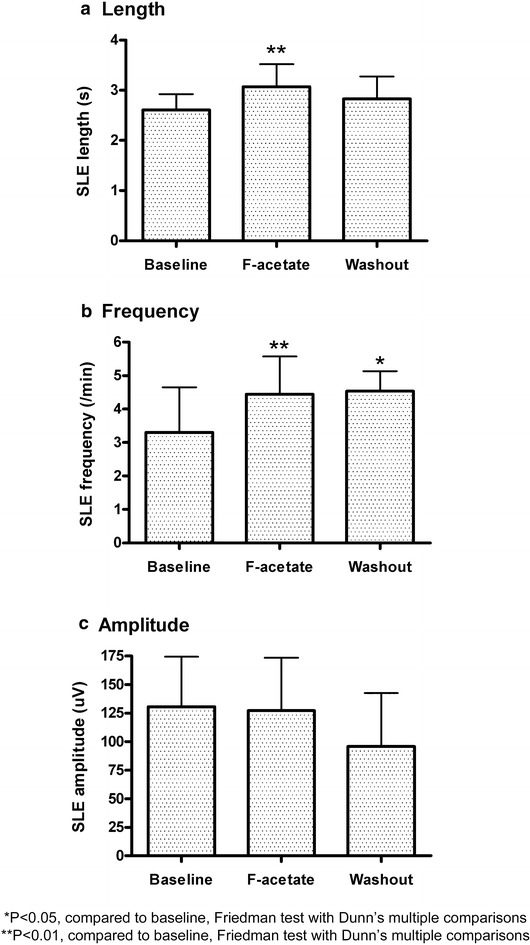
Effect of fluoroacetate (F-acetate) (combined doses 1, 5 and 10 mM) on seizure-like event (SLE) **a** length, **b** frequency and **c** amplitude (n = 17). The data are normalised to the baseline values and are expressed as mean + SEM. *p < 0.05, compared to baseline, Friedman Test with Dunn’s Multiple Comparisons. **p < 0.01, compared to baseline, Friedman Test with Dunn’s Multiple Comparisons

Contrary to the main hypothesis, fluoroacetate enhanced SLE activity, with no clear dose-dependence. SLE length and frequency, respectively, increased on average by 17 and 10 % for the 1 mM dose (n = 3, 1 animal); 24 and 34 % for the 5 mM dose (n = 9, 1 animal, both statistically significant increases); and 15 and 16 % for the 10 mM dose (n = 5, 1 animal). Thus, the trends were similar across all doses. The data were pooled (n = 17, 2 animals) and are illustrated in Fig. 3. The possibility of a time-effect should be noted for SLE frequency (Voss et al. 2014), although the levelling of this parameter during drug washout suggests at least a part drug effect. SLE amplitude was not significantly altered, although the downward trend during washout also hints at the possibility of a time effect.

**Fig. 4:**
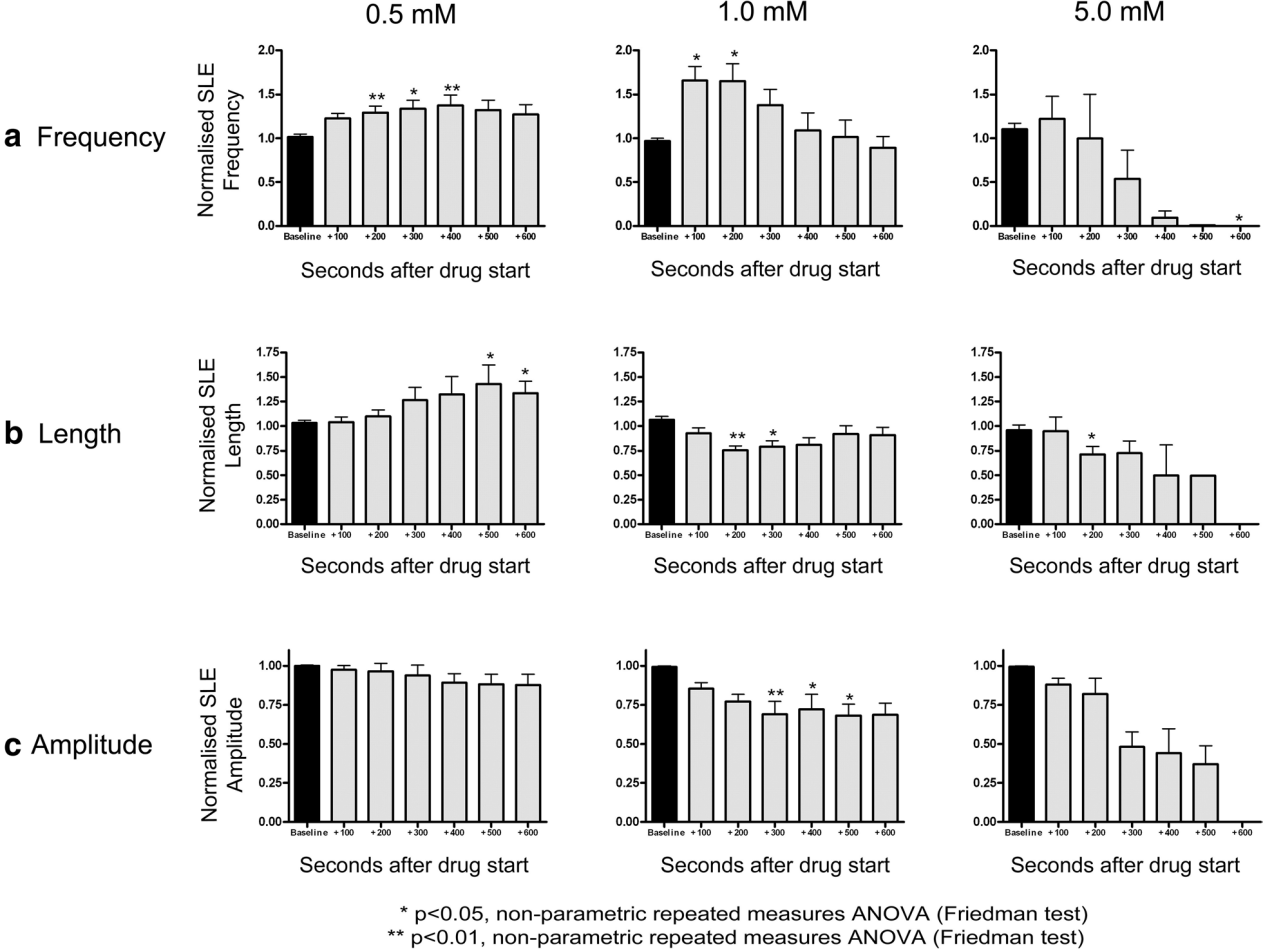
Effect of 0.5 (n = 17), 1.0 (n = 13) and 5.0 mM (n = 8) aminoadipic acid on seizure-like event **a** frequency, **b** length and **c** amplitude. The data are normalised to baseline values and are expressed as mean + SEM. *p < 0.05, compared to baseline, Friedman Test with Dunn’s Multiple Comparisons. **p < 0.01, compared to baseline, Friedman Test with Dunn’s Multiple Comparisons

**Fig. 5:**
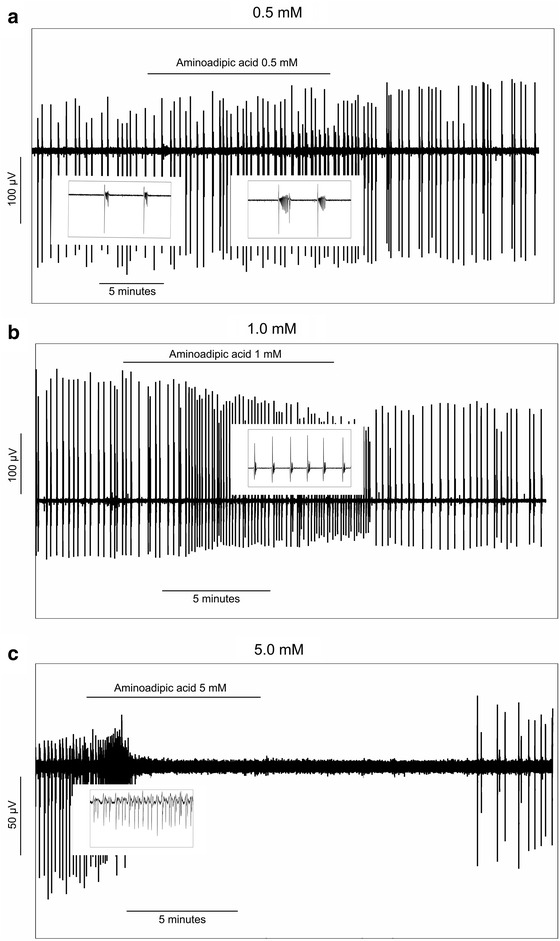
Examples from three slices showing the effect of **a** 0.5 mM, **b** 1.0 mM and **c** 5.0 mM aminoadipic acid on seizure-like event (SLE) characteristics. In each *plot*, the individual *vertical lines* represent single SLE events. The thumbnail inserts show zoomed sections from the corresponding time points in the main figure

The 0.5 mM dose of aminoadipic had an effect similar to that of fluoroacetate, with a significant, reversible increase in SLE frequency and length (see Figs. 4, 5). The 1.0 and 5.0 mM doses had biphasic effects, with an initial excitation (increase in SLE frequency), coupled with a reduction in event length and amplitude. SLE activity was reversibly silenced at 5.0 mM following a brief surge in event frequency. These effects are illustrated in Figs. 4 and 5.

Prolonged perfusion of 1 mM aminoadipic acid for 2 h (n = 3, 2 slices from one animal) consistently and reversibly increased event frequency, with no clear effect on either SLE length or amplitude. Thus, the effect of short (10 min) and prolonged (2 h) aminoadipic acid perfusion were similar.

Aminoadipic acid (1 mM) perfused in normal aCSF did not induce SLE activity in three recordings from two slices. Zero-magnesium perfusion induced robust SLE activity at each of the same three recording locations, confirming that the slices were viable. Thus, while aminoadipic acid had neuroexcitatory effects, it did not induce SLE activity unaided.

### Combined astrocyte inhibition and anaesthetic exposure

In slices pretreated with 0.5 mM aminoadipic acid, the capacity for both propofol (n = 2, 1 animal) and etomidate (n = 2, 1 animal) to strongly reduce SLE frequency remained intact.

### Results summary

The aim of this study was to investigate whether the hypnotic action of NMDA antagonist and GABA agonist general anaesthetics could be explained by cerebrocortical suppression of astrocyte function. To this end, we first sought to identify in cortical slices a robust correlate of in vivo hypnotic potency—and found that the magnitude of the reduction in SLE frequency in slices correlated well with the ability of each test drug to induce hypnosis in vivo. With this as a comparator, we then determined the effect of astrocyte metabolic inhibition on cortical slice SLE activity. We reasoned that if anaesthetic suppression of astrocyte activity mediates the hypnotic action of these drugs, then targeted astrocyte inhibition in slices should be apparent as a reduction in SLE frequency. This was clearly not the case—we observed the opposite effect, with enhancement of SLE activity for both pharmacologically distinct astrocyte inhibitors. Additionally, if astrocyte suppression explains anaesthetic hypnosis, we might expect a profound synergy between astrocytic metabolic inhibitors and the response to propofol and etomidate, which was not observed. Accordingly, our results do not support the hypothesis that suppression of astrocytes is a mechanism of anaesthetic hypnosis. While the experimental design of this study indirectly probed the relationship between anaesthetic effects on astrocytes and anaesthetic hypnosis, the logic is clear—if hypnotic anaesthetic action is robustly associated with suppression of SLEs; and astrocyte inhibition has the opposite effect; then astrocyte inhibition is unlikely to be a major component contributing to anaesthetic hypnosis.

### Excitatory effects of astrocyte inhibition

The mechanism of enhanced population activity following astrocytic inhibition is likely to be multimodal. For the dose range of aminoadipic acid applied in this study, the predominant effects are likely to be inhibition of Na-dependent glutamate re-uptake (McBean 1994; Tsai et al. 1996) and reduced kynurenic acid production (Gramsbergen et al. 1997; Wu et al. 1995). A 65 % reduction in glutamate uptake is seen in cultured rat astrocytes at 0.5 mM aminoadipic acid (Tsai et al. 1996). Kynurenic acid is an endogenous excitatory amino acid receptor inhibitor, meaning reduced production will have a neuroexcitatory effect. Aminoadipic acid 0.5 mM applied to thick (1 mm) neocortical sections for 2 h reduces kynurenic acid production by 60 % (Gramsbergen et al. 1997), an effect specifically mediated by astrocytes (Wu et al. 1995). Aminoadipic acid could also cause astrocyte-dependent neuroexcitation by inhibition of glutamine synthetase activity (mediating the astrocytic conversion of glutamate to glutamine) and by inhibition of gamma-glutamylcysteine synthetase (mediating astrocytic synthesis of glutathione)—however at the dose and duration of exposure in the current study these effects are probably negligible (McBean 1994; Tsai et al. 1996).

The mechanism of effect of fluoroacetate is likely to overlap with aminoadipic acid. When applied to rat hippocampal slices for 1–2.5 h, 1 mM fluoroacetate results in an increase in stimulation-induced overflow of extracellular glutamate, providing an adequate supply of glutamine (glutamate precursor) is maintained (Szerb and Issekutz 1987). The reduction in glutamate up-take by astrocytes is probably due to a reduction in the activity of the Na pump, secondary to tricarboxylic acid cycle inhibition and reduced ATP production (Szerb and Issekutz 1987). The similarity of effects on SLE activity of fluoroacetate and low-dose L-alpha-aminoadipic acid in our study can therefore be explained by similar functional end-points.

### Methodological considerations

Astrocytes are acutely sensitive to general anaesthetics, at concentrations below that necessary to effect significant changes in neuronal activity (Schummers et al. 2008). Collectively, the documented effects are inhibitory, including impaired glutamate uptake (Rath et al. 2008), reduction in calcium signalling (Schummers et al. 2008; Thrane et al. 2012) and reduced glial fibrillary acidic protein staining (Jevtovic-Todorovic et al. 2013). In other words, anaesthetics broadly inhibit astrocytic function. It was upon this basis that we applied metabolic blockers to mimic anaesthetic suppression of astrocyte function.

Interpretation of the findings of this study depends critically on posology. A dose of 45 µg/ml was chosen for all remiketamine variants on the basis of pilot experiments (data not shown) in which this dose was shown to effect changes in SLE parameters similar to that previously investigated for other anaesthetics (Voss et al. 2012). A higher dose than previously used for ketamine (4 µg/ml; Voss et al. 2012) was necessary because the ketamine analogues are rapidly deactivated by tissue esterases (Harvey et al. 2015).

The drug dose ranges for propofol and etomidate were chosen to be clinically relevant, based on previous studies investigating the diffusion characteristics of etomidate into brain slice tissue (Benkwitz et al. 2007) and the relative in vivo potencies of etomidate and propofol (Avramov et al. 1995). Briefly, in 400 µm slices, the etomidate concentration at a depth of 100–200 µm approaches 50 % of the concentration in the bath within approximately 20 min (Benkwitz et al. 2007). For the lowest etomidate dose in our study (8 µM) this would equate to a tissue concentration of 4 µM at the end of the 30 min delivery period. An effect-site etomidate concentration of 2 µM renders 50 % of rats anaesthetised and 4–12 µM equates to deep anaesthesia (De Paepe et al. 1999). The dose ranges for sodium fluoroacetate and aminoadipic acid were based on previous investigations utilising the same drugs in similar in vitro preparations (Benjamin and Verjee 1980; Charles and Chang 1983; Cheng et al. 1972; Haugstad and Langmoen 1997; Saito 1990). At the highest dose of aminoadipic acid (5 mM), a strong inhibition to SLE activity was observed. We believe this was the result of a direct *neuronal* effect, based on four observations:

1. The aminoadipic acid LD50 in *glial* cultures is approximately 0.6 mM (Bridges et al. 1992).
2. In cultured cerebellar cells, aminoadipic acid is toxic to both *neurons* and *glial* cells at 1.5 and 5.0 mM (Garthwaite and Regan 1980).
3. 3 mM aminoadipic acid applied to striatal slices results in *neuronal* degeneration, while no neuronal effect is seen when administered at 1 mM for 40 min (McBean 1990).
4. Aminoadipic acid at 0.5 mM in the present study had effects on SLE activity that were qualitatively identical to that of fluoroacetate (1–10 mM)—while fluoroacetate does not directly affect *neuronal* excitability following 5 mM application to rat cortical slices for 40 min (Fossat et al. 2012).

Our conclusion is that astrocyte-specific effects predominate in the 0.5–1.0 mM dose range for aminoadipic acid and that the excitatory effects seen at 0.5 and 1.0 mM can be interpreted accordingly. The alternate possibility of a weak direct neuroexcitatory effect of aminoadipic acid via glutamate receptors cannot be completely ruled out (McLennan and Hall 1978). Aminoadipic acid is only 15 % as potent as L-glutamate (McLennan and Hall 1978). The similarity of response in the present study between that of fluoroacetate (which does not directly affect neurons) and 0.5 mM aminoadipic acid indicates that a direct neuroexcitatory effect at this low dose is at best negligible.

### Conclusion

In conclusion, the results of this study fail to support the underlying hypothesis that astrocytic inhibition causes anaesthetic hypnosis. A role for astrocytes in limiting neuroexcitation is identified.

## Methods

For the in vivo study, adult Sprague–Dawley rats were obtained from the Ruakura Animal Research Centre, Hamilton, New Zealand, with approval from the Ruakura Animal Ethics Committee. For the in vitro cortical slice study, adult mice (C57/BL6/129SV) were obtained from a breeding colony at Waikato University, Hamilton, New Zealand, with approval from the Waikato Animal Ethics Committee.

The methods were divided into two parts.

### Part 1: correlating in vivo hypnotic potency with cortical slice electrophysiology

#### In vivo analysis of anaesthetic hypnotic potency

The in vivo analysis of the hypnotic potency of 21 ketamine analogue compounds has been reported in part elsewhere (Harvey et al. 2015; Jose et al. 2013). The results form part of a wider screening investigation pursuing the development of ketamine-ester analogues with rapid offset characteristics via hydrolysis of pharmacologically active ester groups. This set of compounds included seven non-ester entities (including ketamine), but for simplicity we will retain the term “ketamine-esters” to describe the collective group. For the purpose of this study, these ketamine-ester variants provided a range of structurally similar compounds with varying hypnotic potencies—which could be correlated with their effect on cortical slice field potential activity.

The methodology of drug design, synthesis and testing has been detailed previously (Harvey et al. 2015; Jose et al. 2013). In brief; adult female Sprague–Dawley rats (n = 3 per agent) were non-traumatically restrained and the marginal vein of the tail cannulated. One of 21 ketamine-ester analogues was delivered at 10 mg/ml via a minibore extension tube secured to the tail. Weight-adjusted infusions were administered at 20 mg/kg/min initially and continued until the animal lost both its ability to maintain righting, and attenuated its withdrawal response to firm digital pressure on the forepaw. Thereafter, the infusion rate was reduced to 6.7 mg/kg/min and adjusted in an up-and-down fashion to maintain dorsal recumbency for 10 min, before cessation. The dose (mg/kg) to loss of righting was adopted as a measure of effective hypnotic potency.

#### Cortical slice electrophysiology

##### Cortical slice preparation

Cortical slices were prepared from adult mice of either sex. The animals were anaesthetised with carbon dioxide prior to decapitation and brain dissection. The cerebrum was placed into ice-cold carbogenated (95 % O_2_; 5 % CO_2_) artificial cerebrospinal fluid (aCSF) containing: 92.7 mM NaCl, 3 mM KCl, 19 mM MgCl_2_, 0 mM CaCl_2_, 1.2 mM NaH_2_PO_4_, 24 mM NaHCO_3_ and 25 mM d-glucose (Nowak and Bullier 1996). Coronal slices (400 µM) were cut between Bregma −1 to −5 mm on a vibratome (Campden Instruments, UK) and transferred to a holding bath with carbogenated aCSF containing zero magnesium (124 mM NaCl, 5 mM KCl, 2 mM CaCl_2_, 1.25 mM NaH_2_PO_4_, 26 mM NaHCO_3_ and 10 mM d-glucose). The slices were left undisturbed for at least an hour prior to recording at room temperature (approximately 28 °C).

##### Electrophysiology recording parameters and experimental procedure

One slice at a time was transferred to a recording bath (Tissue Recording System, Kerr Scientific Instruments, New Zealand) perfused with carbogenated zero-magnesium aCSF at a gravity-fed flow rate of 6.0 ml/min. Removal of magnesium ions from the aCSF activates the cortical tissue, resulting in spontaneous field potential activity resembling short seizure-like events (SLEs) that can be recorded unabated for several hours (Voss and Sleigh 2010). Field potentials were recorded using Teflon-coated (50 µm) tungsten electrodes, referenced to a silver/silver-chloride electrode located in the recording bath. Up to four recording electrodes were positioned equidistant apart in the cerebral cortex, with no particular cortical location targeted. The data was recorded with a 1000× gain, low- and high pass filtered at 1000 and 1.0 Hz respectively (Model 1800 AC amplifier, A-M Systems, USA) and sampled at a frequency of 5000 samples/second (Power 1401, Cambridge Electronic Designs, UK). Recordings were saved for analysis using Matlab (Version 7.3.0.267 (R2006b), The Mathworks Inc., Natick, MA, USA).

##### Testing ketamine variants in cortical slices

Recordings were made from 24 slices from 6 animals. SLE activity was recorded for at least 10 min to achieve a baseline. Thereafter, one of the ketamine-ester test agents was perfused at 45 µg/ml for 20 min followed by drug washout for 20 min with drug-free zero-magnesium aCSF. All agents were tested at the same dose. On eight occasions, two or three agents were tested in the same slice, in which case sufficient time was allowed for SLE activity to return to baseline levels before perfusing the next drug. Where multiple electrodes were positioned in the same slice, each channel was considered an independent recording on the condition that SLE activity was not coupled between locations. The basis of this proviso is that neocortical SLE activity can be generated from multiple independent locations within the same slice, just as if the slice was physically sectioned between recording locations (Voss et al. 2012).

##### Testing propofol and etomidate in cortical slices

In addition to the ketamine variants, dose response characteristics of propofol and etmoidate, two established general anaesthetics, were quantified in cortical slices. For propofol, 28 recordings were made from 20 slices (10 animals) and for etomidate, 18 recordings were made form 14 slices (9 animals). Following at least 10 min of baseline SLE recording, one or other drug was perfused at 3 sequential doses (28, 56 and 84 µM for propofol and 8, 16 and 24 µM for etomidate). Each dose was applied for 30 min in a step-wise manner until either the maximum dose was reached or the SLE frequency reduced to <50 % of that established during baseline. Thereafter, washout with zero-magnesium aCSF was continued for 40 min.

#### Data analysis

For the ketamine-esters, the drug effect on SLE frequency, length and amplitude was quantified as the mean percent change in each parameter from baseline relative to the 15–20 min period towards the end of drug perfusion. This period represents the time at which the drug was at peak concentration within the slice bath, taking into consideration the initial wash-in period. The in vivo hypnotic potency [the dose (mg/kg) to loss of righting] for each variant was related to its effect on SLE frequency, amplitude and length. Linear regression was used to quantify the relationship between in vivo hypnotic potency and change in each SLE parameter.

For the propofol and etomidate experiments, SLE frequency was normalised to baseline and averaged over three 30 min time periods corresponding to each drug dose (offset by 10 min to the start of each dose to allow for drug equilibration in the perfusion bath) and a washout period at the end of the recording. If the second or third dose was not delivered (because SLE frequency had already reduced by more than 50 %), for that recording the frequency was assumed to be zero for the analysis of those time periods.

### Part 2: testing astrocytic metabolic inhibition on cortical slice SLE activity

The methods for cortical slice preparation and electrophysiological recording of zero-magnesium SLE activity were as described above.

Two astrocyte metabolic inhibitors were tested, fluoroacetate and aminoadipic acid. Fluoroacetate was delivered in three concentrations, 1, 5 and 10 mM. The pH of the 5 mM solution was 7.52 and was not adjusted. The pH of the 10 mM solution was 7.74 and was adjusted to 7.58 with 0.1 M HCl. The osmolarity of the 10 mM solution was adjusted to the equivalent of 5 mM by reducing the NaCl concentration in solution by 5 mM. All fluoroacetate concentrations were run for 45 min, followed by drug washout for 40 min. The effects were similar across all concentrations, therefore the data was pooled. For statistical analysis, SLE amplitude, length and frequency were averaged for each slice over three broad epochs: the 7 min period prior to drug delivery; the 45 min period of drug delivery; and the first 20 min period of drug washout. Because the data was not normally distributed (Kolmogorov and Smirnov test), the three epochs were compared statistically using non-parametric repeated measures ANOVA (Friedman test).

Aminoadipic acid was delivered in 3 concentrations, 0.5 (n = 17 from 3 animals), 1.0 (n = 13 from 2 animals) and 5 mM (n = 8 from 2 animals). The pH of the 1 mM solution was 7.54 and was not adjusted. The pH of the 5 mM solution was 7.2 and was adjusted to 7.58 with 0.1 M NaOH for three slices. The effect was qualitatively identical whether pH was adjusted or not and the data was therefore pooled. The 0.5 mM solution was run for 15 min, the 1 mM solution for 10 min and the 5 mM solution for 7 min, followed by drug washout. SLE amplitude, length and frequency were averaged for each slice over seven 100 s epochs, one during baseline recording 3 min before start of drug infusion and six sequential epochs from the start of drug infusion. This enhanced time resolution was necessary because aminoadipic acid had multiple effects both within and between the three concentrations tested. Because the data was not normally distributed (Kolmogorov and Smirnov test), the epochs were compared statistically using non-parametric repeated measures ANOVA (Friedman test). Only statistical comparison to the baseline epoch is reported. In three cases for the 5 mM dose, recording was terminated immediately after SLE activity ceased. It was assumed in these cases that SLE activity would have continued suppressed for the remaining epochs under analysis, in keeping with the data from the slices in which recording was continued.

#### Combined astrocytic metabolic inhibition and anaesthetic delivery

To confirm whether the anaesthetic effect on SLE frequency persisted during astrocytic metabolic inhibition, in two recordings each for propofol and etomidate, slices were pretreated with 0.5 mM aminoadipic acid for 15 min before anaesthetic perfusion (84 and 24 µM, respectively). When SLE frequency had reduced to at least half of the baseline frequency, aminoadipic acid and anaesthetic were washed out with drug-free zero-magnesium until SLE activity returned.
